# Improved Enantioselectivity for Atenolol Employing Pivot Based Molecular Imprinting

**DOI:** 10.3390/molecules23081875

**Published:** 2018-07-27

**Authors:** Andreea Elena Bodoki, Bogdan-Cezar Iacob, Laura Elena Gliga, Simona Luminita Oprean, David A. Spivak, Nicholas A. Gariano, Ede Bodoki

**Affiliations:** 1 Department of Inorganic Chemistry, Faculty of Pharmacy, “Iuliu Haţieganu” University of Medicine and Pharmacy, 12 Ion Creangă St., Cluj-Napoca 400010, Romania; abota@umfcluj.ro (A.E.B.); loprean@umfcluj.ro (S.L.O.); 2 Department of Analytical Chemistry, Faculty of Pharmacy, “Iuliu Haţieganu” University of Medicine and Pharmacy, 4 Pasteur St., Cluj-Napoca 400349, Romania; iacob.cezar@umfcluj.ro (B.-C.I.); gliga.laura@umfcluj.ro (L.E.G.); 3 Department of Chemistry, Louisiana State University, Baton Rouge, LA 70803, USA; dspivak@lsu.edu (D.A.S.); nicholas.gariano13@gmail.com (N.A.G.)

**Keywords:** metal-mediated molecular imprinting, hydrophilic template, atenolol, chiral separation, β-blockers, molecularly imprinted polymers, molecular recognition

## Abstract

In the last few decades, molecular imprinting technology went through a spectacular evolution becoming a well-established tool for the synthesis of highly selective biomimetic molecular recognition platforms. Nevertheless, there is still room for advancement in the molecular imprinting of highly polar chiral compounds. The aim of the present work was to investigate the favorable kosmotropic effect of a ternary complex involving a polar chiral template (eutomer of atenolol) and a functional monomer, bridged by a central metal ion through well-defined, spatially directional coordinate bonds. The efficiency of the chiral molecular recognition was systematically assessed on polymers obtained both by non-covalent and metal-mediated molecular imprinting. The influence on the chromatographic retention and enantioselectivity of different experimental variables (functional monomers, cross-linkers, chaotropic agents, metal ions, porogenic systems, etc.) were studied on both slurry packed and monolithic HPLC columns. Deliberate changes in the imprinting and rebinding (chromatographic) processes, along with additional thermodynamic studies shed light on the particularities of the molecular recognition mechanism. The best performing polymer in terms of enantioselectivity (α = 1.60) was achieved using 4-vinyl pyridine as functional monomer and secondary ligand for the Co(II)-mediated imprinting of S-atenolol in the presence of EDMA as cross-linker in a porogenic mixture of [BMIM][BF_4_]:DMF:DMSO = 10:1:5, *v*/*v*/*v*.

## 1. Introduction

Biological or synthetic receptors selectively recognize their target chemicals based on a combination of weak, short-ranged intermolecular interactions, such as hydrogen bonding, π-π interactions and van der Waals forces; and their selectivity being further refined by additional repulsive steric confinements. Molecularly imprinted polymers (MIPs) able to mimic natural receptors, offer tailored selectivity towards target molecules and better chemical and thermal stability in a simple and cost-effective manner [1]. MIPs have been widely employed for the concentration, separation and analysis of various bioactives, either as stationary phases in chromatography and capillary electrophoresis [2,3,4,5,6], or as recognition elements in chemo- and biosensing [7,8,9]. Within the extensive body of literature, a considerable part focuses on the use of these polymers for chiral analysis [2,7,10,11,12,13] in various pharmaceutical, biomedical or environmental applications. Differentiation between the chiral forms of a molecule (chiral discrimination) is considered the supreme form of molecular recognition. Most often, the needed stereospecific features of MIPs are acquired through the process of non-covalent molecular imprinting using a variety of functional monomers, cross-linkers and porogenic solvents. Even though over the last few decades MIPs have become a well-established analytical tool for the selective recognition and analysis of small molecules, there is still some room for advancement in the molecular imprinting of highly polar compounds and biomacromolecules. Recent attempts at imprinting polar compounds such as polyphenols, e.g., oleuropein [14], polar organic micropollutants, e.g., benzotriazole [15] have been described, especially for sample enrichment applications (SPE adsorbents). The addition of certain additives (hydrophilic functional polymers- oligo- and polyethylene glycol methacrylate [16]; molecular crowding agents—polyethylene glycol; room temperature ionic liquids [17,18]) in the pre-polymerization mixture could improve the overall imprinting efficiency, hydrophilicity, flexibility, morphology and porosity of the resulting polymer. Furthermore, alternative imprinting protocols, such as metal ion-mediated molecular imprinting may further correct some of the observed shortcomings in the molecular imprinting and recognition of polar templates [16].

In the pivot-based or metal ion-mediated molecular imprinting (MMMI) process the metal ions act as a bridge between the functional monomer and the template. The monomers are thus regularly positioned around the template via coordinate bonds restraining the free motion of the species. Consequently, the number of non-specific binding sites decreases, and improved imprinting factors are achieved [19]. Since chiral molecular recognition by MIPs relies on very small energetic differences between the forming transient selector/select and complexes at the polymer’s interaction sites, any improvement in the degree of order throughout the polymerization step is beneficial in conveying the template’s (i.e., target enantiomer) molecular information to the emerging MIP recognition sites with the highest possible fidelity.

Metal pivot-based imprinted polymers designed for water soluble templates were previously reported by the enhancement of column permeability and affinity towards the polar template [16,18,20,21]. However, the number of metal-mediated imprinted polymers intended for chiral separation is rather scarce [16,17,22]. Various representatives of β-blockers, most often propranolol [23,24,25,26], have been employed as model compounds in demonstrating the enantioselectivity of various chiral selectors. Nevertheless, for atenolol (ATNL), as one of the most polar representatives of this class of drugs, usually the poorest enantioselectivity has been reported under optimized binding conditions [26,27,28].

The aim of the present work was to investigate the favorable kosmotropic effect of a ternary complex involving both the polar chiral template (eutomer of ATNL) and the functional monomer, bridged by the central metal ions through well-defined, spatially directional coordinate bonds. Various aspects of the formation of the ternary metal complex monitored by UV-Vis spectroscopy, as well as particularities regarding the molecular imprinting process (composition of pre-polymerization mixture, initiation of free-radical polymerization, etc.) are also discussed.

## 2. Results

### 2.1. Ternary Metal Complexes of ATNL

In order to engage a more rational approach in selecting the appropriate metal ion, functional monomer, molar ratio and porogenic solvent to be tested for subsequent molecular imprinting, UV-Vis spectroscopy provided a simple, fast, cost-effective and relatively straightforward instrumental method which is adjustable to small sample volumes for assessing the formation of the ternary complex. The electronic spectra (350–1100 nm) of binary and ternary complexes of several transition metal ions (Co(II), Cu(II) and Ni(II)) with the ATNL as primary ligand, and various functional monomers (4-vinyl pyridine (4-VPy), 1-vinyl imidazole (VIM), methacrylic acid (MAA), acrylamide (AM), *N*,*N*′-methylenebis(acrylamide) (BAM), *N*,*O*-bismethacryloyl ethanolamine (NOBE), vinyl ferrocene (VFC), 4-vinyl phenylboronic acid (4-VPBA), trans-2-chloromethylvinylboronic acid (CVPBA) and poly(ethylene glycol) methyl ether methacylate (M_n_ = 300 g mol^−1^, PEGMA) prepared in a mixture of DMF/DMSO 1:5 (*v*/*v*) were recorded.

**Figure 1:**
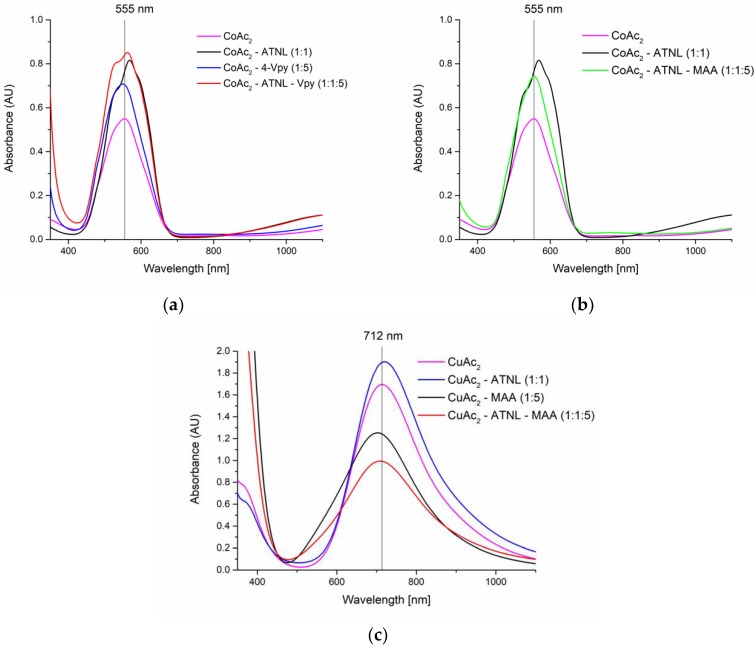
Electronic spectra of Co(II) complexes in DMF/DMSO 1:5 (*v*/*v*) with (**a**) binary and ternary Co(II) complexes with ATNL (1:1 molar ratio) and 4-VPy (1:5 molar ratio); (**b**) binary and ternary Co(II) complexes with ATNL (1:1 molar ratio) and MAA (1:5 molar ratio); (**c**) binary and ternary Cu(II) complexes with ATNL (1:1 molar ratio) and MAA (1:5 molar ratio).

Bathochromic and hypsochromic shifts or hyperchromic and hypochromic effects occurring in the electronic spectra of ternary metal-template-monomer species with respect to the spectra of binary metal-template analogues were interpreted as evidence of ternary complex formation. Co(II) ions were selected as the pivot for the MIPs using 4-Vpy as a monomer (Figure 1a,b), while Cu(II) ions were preferred for the imprinting process using an acidic monomer, MAA (Figure 1c).

### 2.2. Preparation of MIPs

#### 2.2.1. Non-Covalent Molecular Imprinting

Based on the ability of ATNL to interact in a concerted manner with hydrogen-bonding monomers, choices of host functional monomers ranged from the most commonly used donor-acceptor type monomers, such as MAA, AM or VIM to the ones bearing a single hydrogen donor or acceptor motif, such as 4-VPy (Table 1, Table 2 and Table 3). Choices among the conventionally employed aprotic porogenic solvents for non-covalent imprinting (i.e., toluene, ACN) were often limited by the poor solubility of the highly polar template. Thus, the influence of different aprotic (ACN, DMF, DMSO) and protic (methanol (MeOH), butanol) porogenic solvents on the overall features of the resulting imprinted polymer were also studied. Concomitantly, the influence of different additives (i.e., cross-linkers, ionic liquids) meant to balance the polymeric framework’s flexibility and porosity, as well as the selectivity of the imprinted binding sites, were investigated. Both bulk (S4) and monolithic (M6, M16-20) MIPs were obtained using this imprinting approach. Modest to no enantioselectivity (α = 1.0–1.32) was recorded, amongst which the monolithic polymer with 4-VPy as functional monomer (M6, α = 1.30), reticulated with EDMA in the presence of an ionic liquid ([BMIM]BF_4_) in DMF/DMSO 1:5 (*v*/*v*) as porogenic media was considered the most promising candidate for further investigations by the MMMI approach. Comparable results were obtained employing MAA as a functional monomer in ACN as a porogenic solvent (M18, α = 1.32). The presence of a protic solvent such as MeOH or water, even in trace amounts, added to the solvation of the template in ACN compromised chiral recognition (M20, α = 1.00).

#### 2.2.2. One-Monomer Molecularly Imprinted Polymer (OMNiMIP)

In an effort of reducing to a minimum the number of variables involved in the traditional non-covalent molecular imprinting, the OMNiMIP approach introduced by Sibrian-Vasquez and Spivak [29] was also tested by using a single crosslinking monomer, NOBE, in addition to the chiral template, solvent (DMF) and initiator (Table 1). Under these conditions, the obtained enantioselectivity was somewhat below expectations (S1, α = 1.05). However, when using ACN as porogen, in which case the presence of a small amount of MAA for the solubilization of ATNL was necessary, a certain improvement in enantioselectivity of the slurry packed column (S4, α = 1.17) was observed.

#### 2.2.3. Metal Ion Mediated Molecular Imprinting

The protocols of molecular imprinting were adapted in compliance with the requirements of the metal ion-mediated approach. Three transition metal ions (Co(II), Cu(II) and Ni(II)) were employed as coordination centers (Table 1, Table 2 and Table 3). Initially, the use of metal ions alongside or together with the single crosslinking monomer (NOBE) did not provide high enough enantioselectivities (S2, α = 1.05; S3, α = 1.01). Furthermore, the use of different molar ratios of 4-VPy as secondary ligand alongside the cross-linker TRIM and porogenic mixture (DMF/DMSO 1:1, *v*/*v*) provided low chiral discrimination of the slurry-based columns (S6–10, α = 1.03–1.07). However, changing the crosslinker to EDMA, and adjusting the polymerization media by adding an ionic liquid ([BMIM]BF_4_ in DMF/DMSO 1:5, *v*/*v*) resulted in a considerable enhancement in chiral selectivity (S11, α = 1.32). Eventually, switching from slurry-based to monolithic columns (with expected gain in column efficiency), alongside the concerted benefits of using MMMI and ionic liquid, led to the fabrication of the best performing MIP monolith (M2, α = 1.60). The optimal composition of the pre-polymerization mixture (Table 2, M2) turned out to be S-ATNL:Co(II):4-VPy (1:1:6 molar ratio), EDMA as cross-linker (C:M ratio = 1:4), [BMIM]BF_4_ as ionic liquid in DMF/DMSO 1:5 (*v*/*v*) as porogenic system.

#### 2.2.4. Bulk Imprinting vs. MIP Monolith

**Figure 2:**
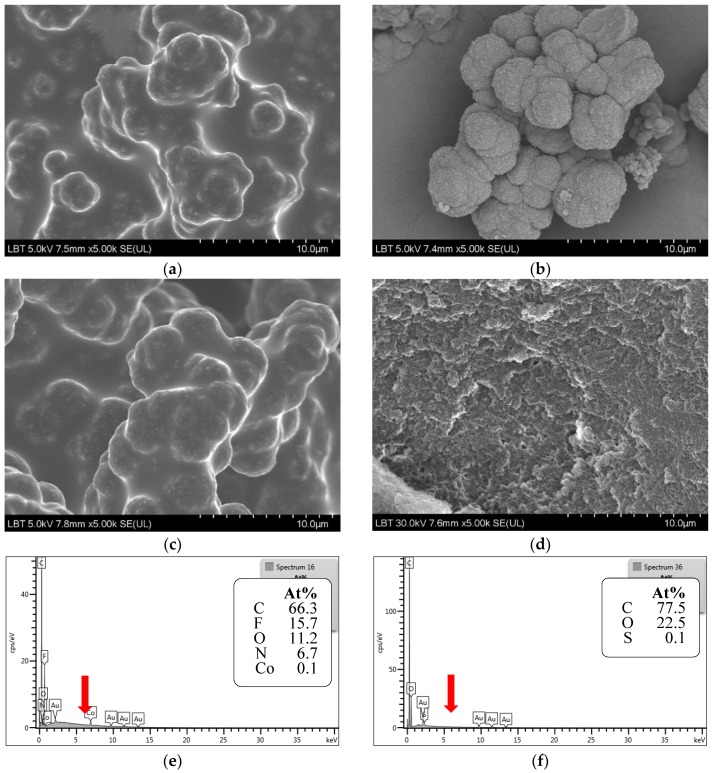
Scanning electronmicrographs of monolithic imprinted polymers of M2 (**a**) before and (**b**) after MeOH:AcOH 9:1(*v*/*v*) washing, (**c**) M8 and (**d**) M5 before MeOH:AcOH 9:1(*v*/*v*) washing. Elemental mapping spectrum of M2 (**e**) before and (**f**) after template removal indicating the washout of the metal pivot ion (Co(II)).

Initially, bulk imprinting with photochemical initialization at lower temperatures was considered (S1–S11, Table 1). After the removal of excess reagents and template (Figure 2a,b), the polymers were ground and sieved, followed by the slurry packing into HPLC columns. Studies continued on monoliths polymerized in situ in the chromatographic column (M1–24, Table 2 and Table 3), but with thermal radical initiation. To further improve the molecular imprinting efficiency and future chromatographic performances of the monoliths, a hydrophilic ionic liquid was also added to the polymerization mixture [18]. Significant differences in the morphology and porosity of the monolith obtained in the presence and absence of the ionic liquid were observed (Figure 2a–d).

### 2.3. Chromatographic Evaluation of the MIPs

**Figure 3:**
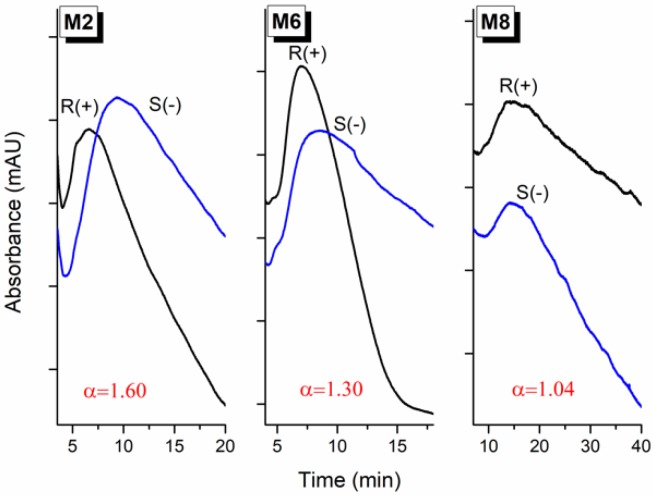
Changes in chromatographic selectivity of metal ion-mediated imprinted (M2), non-covalent imprinted (M6) and non-imprinted (M8) monolithic polymer columns. R(+)—R enantiomer of ATNL, S(−)—S enantiomer of ATNL. For chromatographic conditions see footnote of Table 2.

The enantioselectivity of MIPs investigated as HPLC stationary phases usually exhibits good interassay reproducibility and adequate efficiency with a high sensitivity of detection. Therefore, the present study used standard HPLC columns (100 × 2.1 mm), either slurry packed with the imprinted polymer samples or as a monolith, to study their distinctive chromatographic behavior and recognition mechanism. The assessment and comparison of imprinting factors (IF = k’_MIP_/k’_NIP_) was avoided because the observed binding differences are not exclusively due to the existence of specific imprinted cavities, but also due to the significant differences in the morphology (shape, texture, rigidity, porosity, surface area) of the imprinted (MIP) and non-imprinted polymer (NIP). Thus, the efficacy of molecular recognition was assessed based on the highest enantioselectivity achieved for ATNL’s enantiomers under different chromatographic conditions (mobile phase composition). The results are synthetically presented in Table 1, Table 2 and Table 3, along with some of the most representative chromatograms (Figure 3).

**Figure 4:**
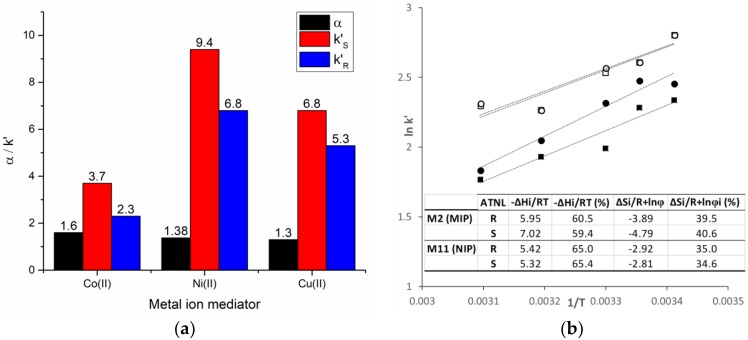
(**a**) Enantioselectivity (α) and chromatographic retention (k’_S_/k’_R_) of metal ion-mediated imprinted monoliths (M2—Co(II), M3—Ni(II), M4—Cu(II)). (**b**) Thermodynamic retention study of ATNL’s enantiomers on metal ion-mediated molecularly imprinted (M2, ●—S-ATNL, ■—R-ATNL) and non-imprinted (M8, ◯—S-ATNL, ☐—R-ATNL) polymers.

To gain better insight into the prevalent chromatographic retention mechanism, the influence of the imprinting approach and polymerization mixture constituents, in addition to the mobile phase composition (organic solvent; ratio, nature and pH of the aqueous buffer) on the polymers’ retention properties were investigated. The nature of the metal ion used in the pivot-based molecular imprinting approach had an important effect on the monolith enantioselectivity (Figure 4a). In 4-VPy-based polymers selectivity is mainly controlled by hydrogen bonding interactions, which are disrupted even by minute amounts of protic solvent (i.e., MeOH, isopropanol, water) added to the mobile phase. In MAA-based polymers, the partition equilibrium is controlled both by ion-exchange and hydrogen bonding interactions, their contribution being dependent on the ratio and pH of the aqueous buffer. Thermodynamic retention studies (Figure 4b) performed on Co(II)-mediated 4-VPy-based imprinted (M2) and non-imprinted (M8) polymers indicate that the binding of ATNL enantiomers involves an important component of the enthalpic (e.g., hydrogen bonding) term.

## 3. Discussion

### 3.1. Ternary Metal Complexes of ATNL

**Figure 5:**
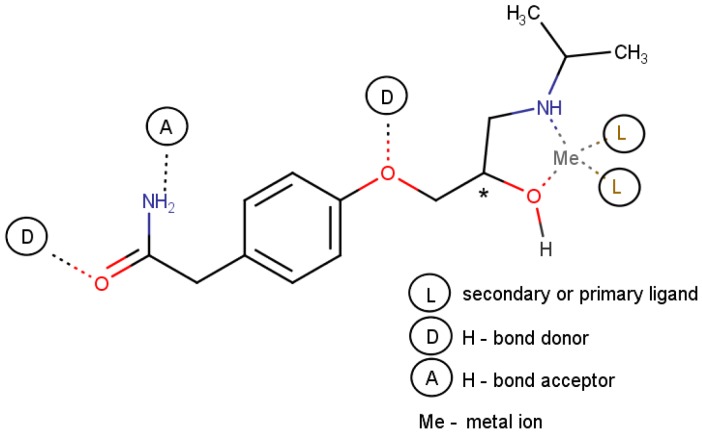
Potential interaction sites (coordinate and/or hydrogen bonding) of ATNL during non-covalent and MMMI.

ATNL, (R,S)2-(4-[2-Hydroxy-3-(isopropylamino)propoxy]phenyl)acetamide (Figure 5), is a beta-adrenergic antagonist, a cardioselective drug with a prolonged effect. Its S(-) stereoisomer exhibits a significantly higher affinity for the β_1_-adrenergic receptors [30]. ATNL is a good chelating agent that may act as a bidentate ligand through the secondary alcohol and amine as electron pair donor moieties and allows the formation of five membered rings that include the central metal ion [31,32,33]. It is also noteworthy that one of the functional groups typically involved in metal ion coordination, the secondary alcohol moiety, is bound to the ATNL’s chiral center. Mononuclear or binuclear binary complexes where the metal to ligand (ATNL) ratio is 1:1, 1:2 or 1:4 have been reported for several first-row transition metal ions (Me = Co(II), Ni(II), Cu(II), Zn(II)) [31,32].

Mononuclear tetrahedral complexes of [Me(ATNL)_2_]^2+^ type with ATNL acting as bidentate, mononuclear octahedral complexes of [Me(ATNL)_4_]^2+^ type [31,34] where two ATNL molecules act as bidentate and the ATNL molecules in the axial position act as monodentate, or an O-bridged binuclear complex, [Cu_2_(ATNL)_2_Cl_2_], where ATNL acts as (O, NH) bridging ligand [33], have been reported for first row transition metal ions (Me = Co(II), Ni(II), Cu(II), Zn(II)).

Ternary complexes of ATNL and ligands with N and O atom donor sites from amine and carboxylic moieties have also been reported for first-row transition metal ions [31,35]. The stability of mixed ligand complexes depends on the characteristics of the approaching secondary ligand (e.g., chelating properties, size and spatial configuration of chelate ring etc.), but also on the possible interactions outside the coordination sphere (hydrogen bonding between coordinated ligands, charge neutralization, chelate effect, and electrostatic interaction between non-coordinated charged moieties of ligands). Ternary complexes of metal ion—primary ligand—secondary ligand in the ratio of 1:1:1, 1:1:2 and 1:2:1, where ATNL was either the primary ligand or the secondary ligand, were obtained. Results indicated the preferential formation of ternary complexes in the 1:1:2 ratio over binary complexes for Co(II) as coordination center [31,35].

Thus, one of the hypotheses of the present study relied on the potential engagement of the hydroxyl group linked to ATNL’s chiroptic center (most likely in its non-deprotonated form [31,33]) in a specific, spatially well-oriented interaction with an appropriate functional monomer mediated by a central metal ion through coordinate bonds. Such a metal ion-mediated molecular self-assembly of the template-monomer in the polymerization mixture should promote beneficial effects in the chiral molecular imprinting process of this hydrophilic enantiomer.

A favorable outcome using MMMI for the synthesis of enantioselective polymers can only occur if in the pre-polymerization step a stable, well-defined and soluble ternary metal complex exists. If in noncovalent imprinting a nonpolar, aprotic porogen, such as toluene or chloroform, is the ideal choice for promoting template-functional monomer associations [19], then in MMMI polar solvents (i.e., DMF, DMSO, MeOH) are required. Polar media may concomitantly offer the prerequisites of a favorable preorganized state (soluble ternary coordination complex): (i) Solvation of the metal ion and polar template and (ii)deprotonation of ligands (both the chiral template and monomer [36]) essential for metal coordination and (iii)dissolution of the resulting ternary metal complex. The stronger and more-defined interactions within the ternary complex are anticipated to lead to more specific recognition sites upon molecular imprinting. Contingent on the nature of the metal ion, its affinity towards the template and the selected monomer and molar ratio of ligands, different ternary metal complexes may also arise. Therefore, the success or failure of MMMI depends on the state of equilibrium established between these coordination complexes.

In the electronic spectra of transition metal complexes d-d transitions, charge transfer transitions, internal ligand transitions, combination and overtone vibrations of the ligands, and intervalence charge transfer transitions, materialize as bands in the region that spans the near infrared, visible and UV region (4000–30,000 cm^−1^). Bathochromic and hypsochromic shifts or hyperchromic and hypochromic effects occurring in the electronic spectra of ternary metal-template-monomer species with respect to the spectra of binary metal-template analogues is an indication of a change in the ligand field environment around the coordination center [37,38,39] for the co-existing species in equilibrium. Based on the data provided by the electronic spectra, Co(II) ions (λ_max_ = 555 nm) were selected as pivot for the MIPs using 4-VPy as a monomer (Figure 1a), while Cu(II) ions were preferred for the imprinting process using an acidic monomer, MAA (Figure 1c).

The electronic spectra of the binary mixtures of ATNL and Co(II), Ni(II) and Cu(II), respectively, recorded in a DMF:DMSO (1:5, *v*/*v*) exhibited relatively weak, low-energy bands in the 13,330–19,230 cm^−1^ (750–520 nm) range which could be assigned to the d-d electronic transitions in a distorted octahedral or tetrahedral coordination environment. The electronic spectra also exhibited higher intensity bands at higher energy regions of the spectra that may be assigned to internal ligand transitions or d–π*, L→M or M→L charge transfer bands. The collected data are in agreement with the bands observed in the electronic spectra of previously reported Co(II), Ni(II) or Co(II) complexes of ATNL [31,32,33].

The d-d electronic transitions translate into two low intensity bands in the electronic spectrum of the binary Co(II)–ATNL mixture, one centered at 17,605 cm^−1^ (568 nm) with a shoulder at 18,868 cm^−1^ (530 nm) and the other at a lower energy region 9090 cm^−1^ (around 1100 nm) (Figure 1a). Upon addition of 4-VPy to the binary mixture, the local coordination environment around the Co(II) ion changes, and this translates into a broader multiple structured band centered at 18,348 cm^−1^ (545 nm) that appears in the spectra of the ternary mixture. The hypsochromic shift of the bands is associated to a hyperchromic effect for the shoulder initially at 18,868 cm^−1^ (530 nm) and to a hypochromic effect for the band initially at 17,605 cm^−1^ (568 nm) (Figure 1a).

Nevertheless, the spectra of binary mixtures of Cu(II)–ATNL and Ni(II)–ATNL changes to a much lesser extent upon the addition of 4-VPy (data not shown). In these cases, it appears that the primary ligand, ATNL, gives rise to a more stable binary complex and that the local environment around Cu(II) and Ni(II), respectively, is significantly less influenced by the presence of the secondary ligand (data not shown).

Acidic monomers such as MAA interact with the Brönsted-basic template ATNL, and the protonation of the amine moiety may alter the beta-blocker’s chelating properties. Such a phenomenon was observed in case of the binary Co(II)-ATNL mixture. The local environment around the Co(II) ion changes significantly upon addition of MAA. As shown by the electronic spectra, ATNL is apparently displaced from the coordination sphere of Co(II) (Figure 1b). In contrast, the electronic spectra indicates no alteration of the local environment around Cu(II) when the acidic monomer is added to the binary Cu(II)–ATNL binary mixture (data not shown).

### 3.2. Preparation of MIPs

#### 3.2.1. Non-Covalent Molecular Imprinting

ATNL has several hydrogen donor and acceptor atoms (Figure 5) which during the non-covalent molecular imprinting could interact in a concerted manner with hydrogen-bonding monomers. Choices of host functional monomers ranged from the most commonly used donor-acceptor type monomers, such as MAA, AM or VIM to the ones bearing a single hydrogen donor or acceptor motif, such as 4-VPy. Furthermore, being a Brönsted-basic template, during the ATNL interaction with MAA a partial or full proton transfer is expected to occur. Based on the nature of the employed porogen, contact hydrogen bonded assemblies may be formed in aprotic solvents such as ACN. Nevertheless, polar protic solvents tend to disrupt such electrostatic interactions having a negative impact on the imprinting factors, but also on the rebinding mechanism of the resulting imprinted material [40]. Different additives (i.e., cross-linkers, ionic liquids [36]) meant to balance the polymeric framework’s flexibility and porosity, as well as the selectivity of the imprinted binding sites, were used in the pre-polymerization mixture (Table 1, Table 2 and Table 3). Modest to no enantioselectivity (α = 1.0–1.32) was recorded, both for the slurry-packed and monolithic columns. Monoliths with different functional monomers (i.e., 4-VPy, MAA) and significantly different porogenic media (DMF/DMSO, ACN) were able to provide similar enantioselectivities (M6, α = 1.30; M18, α = 1.32), as long as the cross-linker and ionic liquid were identical in the polymerization mixture. The ratio of monomer to cross-linker seems to be of less importance in this imprinting approach; however, the presence of a protic solvent such as MeOH or water, even in trace amounts, can compromise the polymer’s enantioselectivity for the hydrophilic template. The non-covalent imprinting of ATNL’s enantiomer by the OMNiMIP approach, at least under the tested experimental conditions, failed to provide noteworthy results in chiral chromatographic selectivity. One of the possible reasons for the low enantioselectivity recorded in the case of the NOBE-based columns may be the significant swelling effect observed during the sieving of the crushed polymer that may affect the structural integrity of the imprinted cavities.

#### 3.2.2. Metal Ion-Mediated Molecular Imprinting

Particular requirements are to be met for MMMI in terms of components of the pre-polymerization mixtures: polar porogenic solvent (DMF, DMSO, DMF/DMSO) that must provide the solubilization of the metal ion’s salt (anhydrous acetates) while keeping the ternary complex in solution, and metal ions that should not compromise the efficiency of the employed free radical initiator (AIBN). Another concern when imprinting polar templates is the chemical and mechanical structure of the polymeric network. A good balance between the polymer’s hydrophilicity and its flexibility determined by the nature of the functional monomer and cross-linker must be optimized. Various additives (i.e., cross-linkers, co-monomer, ionic liquids, chaotropic agents) affecting the prototropic forms or the basicity of participating ligands (ATNL enantiomer and functional monomer) may also be decisive in the formation of the ternary metal complex; therefore, any change in the pre-polymerization mixture had to be carefully considered (Table 1, Table 2 and Table 3).

##### Metal Ion and Functional Monomer (Secondary Ligand)

Following the effect of the nature of metal ion on the efficiency of pivot-based imprinting using 4-VPy as a secondary ligand; results show a decrease in the enantioselectivity of the resulting monoliths in the order Co(II) ≫ Ni(II) ≥ Cu(II) (α = 1.60, 1.38 and 1.30, respectively for M2, M3 and M4 respectively). These findings are correlated with the electronic spectra of the metal complexes recorded in the screening step (Section 2.1) that suggest a higher stability of the Co(II) ternary complex (Figure 1a). Since most of the metal ions from the polymeric framework are eliminated during the template removal process (Figure 2e,f), they are no longer involved in the molecular recognition during rebinding. Nevertheless, the chromatographic retention (k’) is inversely correlated with the recorded enantioselectivities (Figure 4a); this is most probably due to the higher number of non-specific binding sites emerging when Ni(II) and Cu(II) are employed as mediators. Obviously, changing the nature of the secondary ligand is also critical for the chromatographic performance of the resulting chiral stationary phases (CSP). Keeping Co(II) as a pivot and using 1-VIM as a basic secondary ligand bearing the same electron donor moiety as 4-VPy; selectivity decreases to 1.43 (M14). Evidently, in principle, other metal ion–secondary ligand combinations could equal and possibly surpass the selectivity recorded for the S-ATNL:Co(II):4-VPy (1:1:1)-based monolith. Selecting the “right” metal ion-ligand pair is a matter of a rational choice where the number of potential combinations can be significantly narrowed down by a preliminary spectroscopic screening. Therefore, other potentially promising combinations of metal-secondary ligands (Cu(II)–MAA, Cu(II)–1-VIM, Ni(II)–1-VIM) using various polymerization mixture constituents (cross-linkers, porogenic solvent, with and without ionic liquid) were also tested for the imprinting of S-ATNL, but in all cases suboptimal enantioselectivities were recorded (M10, M15, M21–24, α = 1.00–1.14) as compared to the best performing M2 column (α = 1.60). Unfortunately, even if UV-Vis spectroscopy is able to indicate some promising metal-secondary ligand combinations as starting points, assessing the optimal MMMI conditions is far from being a straightforward process. In addition to the formation of the best stable and soluble ternary metal ion-mediated complex, all the other constituents of the polymerization mixture will collectively play a critical part in the final chromatographic outcome, namely enantioselectivity.

It must be stressed that in the absence of the metal ion (i.e., Co(II)), molecular imprinting is achieved by the conventional non-covalent approach, with a poorer performance in terms of molecular recognition (M6, α = 1.30).

##### Functional Co-Monomers

Other co-monomers (i.e., AM, BAM, 4-PBA, CVPBA) added alongside the secondary ligand (4-VPy) had a negative effect on enantioselectivity (S9–10 and M11–13, α = 1.00–1.29). The interaction between co-monomers may reduce to a large extent the binding interactions with the template [41] and may also increase the heterogeneity of the resulting binding sites. Adding hydrophilic macromonomers to the polymerization mixture was reported as another convenient strategy to enhance the imprinting factor of polymers in case of water soluble templates [16]. In our case, instead of further boosting the hydrophilicity of the polymeric network, the addition of poly(ethyleneglycol) methyl ether methacrylate (PEGMA) fully compromised enantioselectivity (M7, α = 1.00).

##### Cross-Linker

Using EDMA instead of TRIM as a cross-linker turned out to be decisive for a more favorable polymer morphology and improved chiral selectivity, regardless of the type of column used (slurry packed–S10, α = 1.07 and S11, α = 1.32; monolith, M1, α = 1.00 and M2, α = 1.60). The beneficial effect of the ionic liquid on the imprinting efficiency was also unequivocally demonstrated by the selectivity value of column M2, α = 1.60 vs. column M5, α = 1.08. Moreover, chromatographic retention is inversely correlated with the recorded selectivity factors in the presence (M2, k’_S_ = 3.7) and absence M5, k’_S_ = 6.3) of [BMIM]BF_4_ most probably due to the increased stability of the ternary complex.

##### Ionic Liquid

The presence of the ionic liquid translates into a spectacular change in the morphology of the imprinted material. A highly porous, globular polymeric framework (Figure 2b, M2) is obtained in the presence of the ionic liquid, while a much denser structure is observed in its absence (Figure 2d, M5). Before template removal, a film-like structure of excess reagents covered the outer surface of the polymer (Figure 2a, M2); while upon washing with MeOH:AcOH 9:1 (*v*/*v*) a highly indented surface was revealed (Figure 2b, M2).

#### 3.2.3. Bulk Imprinting vs. MIP Monolith

MIPs are frequently prepared by bulk polymerization, in which case the obtained polymer block is ground and sieved before use. Bulk imprinting is more suitable for photochemical initiation at lower temperatures, which in theory should provide more homogenous and better defined imprinted sites. However, obtaining CSPs using this approach is cumbersome, wastes large amounts of material, and is often accompanied by the physical damage of some of the binding cavities. Furthermore, the resulting highly irregular MIP particles with polydisperse granulometry dramatically reduce the efficiency of the HPLC columns packed with such materials [42]. Imprinted monoliths polymerized directly in the chromatographic column offer a much simpler and long known alternative [43], at the cost of being only compatible with thermal radical initiation and only amenable to a few porogens. Nevertheless, such a continuous, but highly porous polymeric bed should in principle provide lower backpressure and greatly reduced mass transfer resistance, and thus higher chromatographic efficiency in comparison with the packed MIP-based columns. Room temperature ionic liquids (RTILs), demonstrating excellent solvation features, represent a more environment-friendly alternative as solvents for the preparation of MIPs [44]. As a result of their particular structure, they tend to promote self-assembly and improve specific template–monomer interactions, thus limiting non-specific binding [44,45]. In addition to offering higher imprinting factors and faster polymerization rates, they seem to reduce polymer swelling and boost the permeability of imprinted polymers, including monoliths [46]. Thus, in the search for the optimal experimental conditions that provide the highest enantioselectivity for ATNL’s enantiomers, in our study both imprinting approaches were tested, in the presence or absence of RTIL.

Initially anticipating better molecular imprinting results, bulk imprinting with photochemical initialization at lower temperatures was investigated. Upon polymer grinding and sieving (particle size 25–38 μm) it was expected that all slurry-packed particulate columns demonstrate similar properties in terms of flow, back-pressure and sample load. Given the simplicity and earlier success of the OMNiMIP approach in chiral separation [47], N,O-bismethacryloyl ethanolamine (NOBE) was tested as a single cross-linking monomer for the imprinting of ATNL’s enantiomers. At first (S1-3, Table 1), pure DMF was selected as porogenic solvent which enabled the solvation of the template enantiomer (S-ATNL). Unfortunately, neither the non-covalent (only NOBE), nor the metal ion-mediated (NOBE/Cu(II); NOBE/Co(II) imprinting approach gave notable enantioselectivity (α < 1.04) in any of the tested mobile phases. Nevertheless, adding a small amount of MAA to the NOBE-based polymerization mixture (S4, Table 1) enabled the solubilization of the template in acetonitrile (porogen), and endowed a certain degree of enantioselectivity (α < 1.17) of the resulting MIP using acetonitrile/formate buffer, pH 3.0 (85:15, *v*/*v*) as mobile phase. Seeking for improvements in the chiral recognition of the synthesized polymers, other functional monomers able to form ternary metal complexes with ATNL’s enantiomer were screened by UV-Vis spectroscopy. One promising monomer candidate, forming soluble ternary metal (Cu(II), Co(II), Ni(II)) complexes with the target enantiomer, is 4-VPy (Figure 1). Therefore, different polymerization mixtures (S6–10, Table 1) combining various molar ratios of the template (T), monomer (M), metal ion (Me) and TRIM as cross-linker (C) were screened by metal ion-mediated bulk imprinting using photochemical initiation in a mixture of DMF:DMSO (1:1, *v*/*v*) as porogenic solvent. Unfortunately, none of the slurry-packed columns filled with S6-10 imprinted polymers exhibited noteworthy chiral discrimination (α = 1.00–1.04).

Seeking higher chromatographic efficiencies potentially able to distinguish between the enantiomers of ATNL, studies continued on monoliths polymerized in the chromatographic column using the same molecular imprinting approaches (M1–24, Table 2 and Table 3), but with thermal radical initiation. The polymerization mixture was further adapted, exploiting the favorable influence of RTILs on the imprinting process and on the future chromatographic performances of the resulting continuous polymeric bed. Therefore, a hydrophilic ionic liquid, namely 1-butyl-3-methylimidazolium tetrafluoroborate ([BMIM][BF_4_]), was added to the porogenic solvent system. A higher ratio of IL/porogenic solvent system seem to favor the imprinting efficiency; thus finally a mixture of [BMIM][BF_4_]:DMF:DMSO = 10:1:5, *v*/*v*/*v* was selected [16,17]. Moreover, TRIM was replaced by a more polar cross-linker bearing numerous hydrogen bond acceptor motifs, namely ethylene glycol dimethacrylate (EDMA). Eventually, derived from the tested bulk imprinting polymerization mixtures, keeping a molar ratio of S-ATNL:Co(II):4Vp = 1:1:5 and EDMA as cross-linker (molar ratio M:C = 1:4) resulted a MIP monolithic column (M2) offering the best recorded selectivity factor (α = 1.60) in pure acetonitrile as mobile phase. Interestingly, in the absence of [BMIM][BF_4_] the resulting MIP monolith, (M5) is fully deprived of enantioselectivity (α = 1.00). Yet again, without the Co(II) ion mediating the interaction between the template and monomer during the radical polymerization, the enantioselectivity of the imprinted monolith (M6) drops to 1.30. Nevertheless, since a certain degree of chiral selectivity is still preserved by the M6 monolith, this suggests that in the current experimental conditions a mixed mechanism of imprinting (both non-chiral and metal ion mediated) is most likely to occur in case of M2 monolith. Keeping the same molar ratio of T:Me:M = 1:1:5 as for M2, the enantioselectivity of the resulting MIP monoliths for Ni(II) and Cu(II) (M3 and M4) were also investigated. Although in these cases a certain degree of selectivity for the template enantiomer has been observed (α = 1.38 for M3 and α = 1.30 for M4), it only matched the selectivity factor registered for the monolith obtained in the absence of the metal pivot (M6). The recorded changes in the electronic spectra of the binary and ternary mixtures of S-ATNL-Me(II)-4-VPy display a tendency in the formation of a stable ternary complex in the order Co(II) > Ni(II) > Cu(II) (data not shown). The latter trend correlates well also with the recorded efficiency of chiral recognition for monoliths M2–4. For the control experiments, the reference, non-imprinted monolith (M8) prepared in the absence of the template, but in the presence of Co(II), did not demonstrate any noticeable enantioselectivity (α = 1.04). Evidently, no enantioselectivity is observed for the non-imprinted monolith in the absence of the metal mediator (M9, α = 1.00).

### 3.3. Chromatographic Retention Mechanism

The influence of the mobile phase composition on the polymers’ retention properties, regardless of the employed functional monomer (MAA, 4-VPy) or imprinting approach, indicated a severe decrease of the column capacity factor for both enantiomers of ATNL upon the addition of protic solvents, such as MeOH, acetic acid, or aqueous buffers (data not shown). This would suggest that hydrogen bonding plays a major role in molecular recognition of ATNL’s enantiomers on the tested MIPs.

When using monomers with ionizable functional groups, such as MAA (M17–18), a relatively good efficiency with modest selectivity (α = 1.15–1.32) may be achieved in mixed aqueous(pH ≤ 5)-organic mobile phases (i.e., ACN: 50mM formate buffer (pH 3.0) = 85:15, *v*/*v*) where the partition equilibrium is most probably controlled by ion-exchange interactions (both specific and non-specific) between the amine of the template and the carboxylic groups of the imprinted polymeric structure. Nevertheless, using pure ACN (aprotic, weak solvent with intermediate polarity) as mobile phase, ATNL is totally retained by the polymer due to the synergistic effect of both the electrostatic and the additionally emerging hydrogen bonding interactions.

In the case of the 4-VPy-based MIPs (S6–11; M1–13, Table 1, Table 2 and Table 3), the ion-exchange mechanism is absent, thus retention and selectivity are mainly controlled by hydrogen bonding interactions. As already mentioned, using 4-VPy as functional monomer, enantioselectivity (where applicable) is only recorded in pure ACN. Chromatographic retention of ATNL drops dramatically (k’~1.05) and is accompanied by a complete loss of selectivity upon the addition in the mobile phase of minute amounts (0.1%, *v*/*v*) of protic solvents able to compete with hydrogen bonding.

Unfortunately, upon addition of the porogenic system to the mobile phase, the “memory effect” of the tested polymers could not be improved in the current experimental setup. Due to its strong molar absorptivity, even 1% (*v*/*v*) of DMF:DMSO 1:5 mixture added to ACN hindered the UV signal of the eluting enantiomers (data not shown).

Furthermore, the thermodynamics of the molecular recognition were assessed on the best performing 4-VPy-based monolithic polymer (M2). The retention and sorption selectivities were studied in the temperature range 20–50 °C at a flow rate of 0.2 mL min^−1^ ACN. The influence of temperature on the retention (k’) of enantiomers is shown in Figure 4.

As expected, the experimental van’t Hoff plots recorded for the MMMI polymer (M2) for both enantiomers of ATNL are linear (r^2^ > 0.91), showing decreasing retention with the increase of temperature. The average value of the thermodynamic terms (−ΔHi/RT and ΔSi/R + lnφ) [48] for the two enantiomers were also calculated for the studied temperature range (Figure 4 inset). For the imprinted polymer, the percent contribution of the entropic term (ΔSi/R + lnφ) for both enantiomers lays around 40%. The binding of ATNL enantiomers implies more selective energetic interactions with the M2 monolith, rather than the entropically controlled steric interactions with the imprinted memory sites [49,50]. The average percent contribution of the steric term (ΔSi/R + lnφ) for the binding of the template is slightly higher (40.6%) in comparison with the binding of its antipode (R-ATNL, ~39.5%). In case of the non-imprinted polymer (M8), identical van’t Hoff plots (r^2^ > 0.86) were obtained for both enantiomers. For this polymer, the average steric term contribution for the template and its antipode are somewhat smaller, 34.6% and 35%, respectively. These results indicate that energetic interactions (hydrogen bonding) are mainly responsible for recording the chromatographic retention, but the steric complementary (shape and size) of the emerging imprinted cavities also brings their contribution to the selective binding of the ATNL enantiomers in comparison with the reference, the non-imprinted polymer.

## 4. Materials and Methods 

### 4.1. Reagents

Analytical grade standard S(-)-ATNL 98% was purchased from Toronto Research Chemicals (Toronto, Canada). R(+)-ATNL 99% was provided from Santa Cruz Biotechnology, Inc. (Dallas, TX, USA). 2-(trifluoromethyl)acrylic acid 98% (TFMAA) and vinylferrocene 97% (VFC) were purchased from Tokyo Chemical Industry (Tokyo, Japan). Methacrylic acid (MAA) 99%, pentaerythritol triacrylate (PETRA), pentaerythritol tetraacrylate (PETEA), trimethylolpropane trimethacrylate (TRIM), 2,2′-azobis(2-methylpropionitrile) 98% (AIBN), 4,4′-azobis(4-cyanovaleric acid) 98% (ACVA), 4-vinylpyridine 95% (4-Vpy) and 1-vinylimidazole 99% (1-VIM) were purchased from Aldrich (Steinheim, Germany). Tetrabutylammonium hexafluorophosphate 98% (4BA6FPh) was provided from Fluka (Steinheim, Germany). Ortho-phosphoric acid 85% (*w*/*w*), glacial acetic acid 100%, hydrochloric acid 37% (*w*/*w*), dimethylformamide 99% (DMF) and ammonium hydroxide 25% (*w*/*w*) pro analysi were purchased from Merck (Darmstadt, Germany). Formic acid 95% (*w*/*w*), 1-butyl-3-methylimidazolium tetrafluoroborate 98% ([BMIM][BF_4_]), acrylamide 99% (AM), *N*,*N*′-Methylenebis(acrylamide) 99% (BAM) and boric acid were obtained from Sigma-Aldrich (Steinheim, Germany) and sodium hydroxide 99.3% from Lach-Ner (Neratovice, Czech Republic). HPLC grade solvents (acetonitrile (ACN), methanol (MeOH), butanol (BuOH), were obtained from Sigma-Aldrich (Steinheim, Germany) and used without further purification. Anhydrous Co(II) acetate 98%, Cu(II) acetate 98% and Ni(II) acetate 99% were purchased from Alfa Aesar (Kandel, Germany) and dimethyl sulfoxide 99.5% (DMSO) was from Carl Roth (Karlsruhe, Germany).

NOBE was synthesized by a previously published method [29].

All other chemicals were analytical reagent grade and were used as received.

Ultrapure water (18.2 MΩ, Barnstead EASYPure ROdi) was used for the preparation of all samples, buffers and related aqueous solutions. Phosphate buffer at various pHs was prepared by dissolving phosphoric acid in ultrapure water and adjusted accordingly with NaOH (1 M).

Stock solutions of 2 mg mL^−1^ ATNL enantiomer were prepared in 2 mL volumetric flasks using ACN as solvent and were stored in the refrigerator at +4–6 °C.

### 4.2. Apparatus

Chromatographic experiments were performed with an Agilent 1200 HPLC system (Agilent Technologies, Waldbronn, Germany), equipped with a degassing unit, quaternary pump, autosampler, column oven and a diode-array detector. Signal acquisition and data processing were performed in the Chemstation B03.01 (Agilent Technologies, Waldbronn, Germany) software. The detection was performed at 200 nm and the flow-rate was 0.2 mL min^−1^. All the mobile phases were filtered through a 0.22 μm membrane filter from Millipore before use. Standard samples of atenolol enantiomers (30 μg mL^−1^) dissolved in HPLC grade ACN were injected in a volume of 10 μL.

The UV-Vis spectra were recorded in conventional (1 cm optical path) quartz cuvettes by a double-beam SPECORD^®^ 250 PLUS (Analytik Jena, Jena, Germany) spectrophotometer in the range of 350–1100 nm. The electronic spectra of each ligand and the colored metal complexes were measured in the same solvent as the one used for the MIP preparation.

### 4.3. MIP Preparation

The molecularly imprinted and the non-imprinted polymers were synthesized under the conditions illustrated in Table 1, Table 2 and Table 3. The pre-polymerization mixtures intended for non-covalent imprinting were prepared by weighing the solid components, followed by the addition of liquid monomers and the adequate solvent(s). In the case of metal ion-mediated imprinting, the anhydrous metal ion salts (acetates) were dissolved in the adequate dry solvent or solvent mixture and ATNL was added to the solution. Subsequently, functional monomers, cross-linkers, ionic liquid and the free radical initiator were admixed. In a typical radical polymerization, 15 mg of AIBN has been employed, whereas in the OMNiMIP approach 24 mg of the same radical initiator has been added. The pre-polymerization mixture was sonicated for 15 min and further degassed by purging a gentle flow of nitrogen for 5 min. Depending on the desired form of the polymer (bulk or monolith), the vials or stainless-steel HPLC columns filled with the pre-polymerization mixtures were sealed and caped. In the case of bulk polymers, photopolymerization was performed both at room temperature (22 ± 2 °C) under a UV lamp for 24 h, in 5 mL sealed glass vials, whereas thermal polymerization was carried out in the BMT Ecocell convection oven at 60 °C for 24 h in 5 mL vials or stainless-steel HPLC columns. Removal of the template was achieved by Soxhlet extraction with MeOH/acetic acid (9:1, *v*/*v*), for 24 h. The polymers were then ground using a laboratory mortar and pestle and then sieved using standard testing sieves (φ = 200 mm, CISA sieves, aperture 38 and 25 μm, respectively), and the fraction between 25 and 38 µm was collected. The particles were slurry packed into stainless-steel columns (length 100 mm, internal diameter 2.1 mm) using an LC-10AT VP pump (Shimadzu, Japan) to full volume for HPLC analysis.

The imprinted monoliths were prepared by in situ thermopolymerization, in stainless-steel HPLC columns (length 100 mm, internal diameter 2.1 mm) sealed with Teflon tape and screwable caps, and kept at 60 °C for 24 h in a BMT Ecocell oven. The removal of the template was achieved by washing with MeOH/acetic acid (9:1, *v*/*v*). Control polymers were synthesized under the same conditions in the absence of the template.

### 4.4. Microscopic Characterization of MIPs

For scanning electron microscopy (SEM) characterization, the polymers (MIP and NIP) were metalized with gold in a Polaron E–5100 plasma-magnetron sputter coater (Polaron Equipment Ltd., Watford, UK) in the presence of argon (45 s at 2 kV and 20 mA). Ultrastructural images were obtained in a FEI Quanta 3D FEG scanning electron microscope (FEI, Hillsboro, ON, USA)) at 30kV and different magnification powers.

### 4.5. Chromatographic Evaluation of The Imprinted Polymers

#### 4.5.1. Bulk MIP

The template was removed from the imprinted polymers by Soxhlet extraction with MeOH:acetic acid (90:10, *v*/*v*) for 24 h. After grinding and sieving (25 and 38 μm), the polymer particles were slurry packed using a Shimadzu LC-10AT HPLC pump into steel columns (100 × 2.1 mm) to full volume for chromatographic experiments. As mobile phase various solvent systems were tested isocratically at 20 °C, at a flow rate of 0.2 mL min^−1^, starting with pure ACN and gradually switching to mixtures of ACN with increasing ratio of aqueous buffers (50 mM formate buffer (pH 3.0); 50 mM acetate buffer (pH 5.0) and 100 mM borate buffer (pH 9.3)).

#### 4.5.2. MIP Monolith

The template and excess reagents were removed from the imprinted polymer monolith by pumping through the column MeOH:acetic acid (90:10, *v*/*v*) at a flow rate of 0.2 mL min^−1^ for 24 h. The columns were equilibrated with the corresponding mobile phase for 12 h at a flow rate of 0.20 mL min^−1^ to remove any remaining template. If not otherwise stated, HPLC analyses were performed isocratically at 20 °C, at a flow rate of 0.2 mL min^−1^ using pure ACN or a mixture of ACN/aqueous buffer in variable proportions as the mobile phase, monitoring the eluted analytes at a wavelength of 200 nm.

For all imprinted polymers the separation factor, α, was measured as a ratio of capacity factors k’_S enantiomer_/k’_R enantiomer_, with k’ determined by the following relation: k’ = (t_r_ − t_0_)/t_0_, where t_r_ is the retention time of the analyte and t_0_ is the retention time of the void volume measured using acetone as marker.

## 5. Conclusions

Improved chiral selectivity of the important β-blocker atenolol was achieved by the addition of a metal pivot which gave an imprinting factor of 1.60 versus the traditional molecular imprinted polymer formulation without the pivot which gave an imprinting factor of 1.32. Atenolol is a hydrophilic drug, and molecular imprinting in polar and/or aqueous phases is difficult for traditional molecular imprinting methods based on non-covalent hydrogen bonding or electrostatic complexes between monomers and template, due to disruption of the complex by the polar/aqueous porogenic solvent. The use of a metal to overcome this complex disruption in polar solvents and to coordinate the template and functional monomer led to approximately 23% improvement in chiral selectivity when using Co(II), and a 16% improvement when using Ni(II). This has an important impact due to the large demand for imprinting templates that are only soluble in highly polar and/or aqueous-based solvents. Furthermore, a 25% enhancement in enantioselectivity was found when using monolithic materials versus ground and sieved bulk imprinted polymers. Investigation of binding parameters showed that better selectivity was not a result of increased binding affinity (i.e., larger k’ values) versus non-metal systems but was due to increase in differential enthalpic contributions of binding between the imprinted polymer and each enantiomer of atenolol. Thus, it can be concluded that the underlying mechanism of improvement of enantioselectivity of the imprinted polymer is due to the metal pivot approach maintaining fidelity of the imprinted site during polymerization. The choice of functional monomer was shown to be important based on the affinity of the functional monomer for the metal-pivot; in particular, 4-vinylpyridine or vinylimidazole did not disrupt important template-metal interactions whereas methacrylic acid displaced at least one of the template-to-metal interactions. This may be general for metal-pivot systems that require close proximity of the template to the metal for creating a selective binding site. In addition, the choice of crosslinker was important for optimum performance, for example, entry M2 shows that an EDMA crosslinked molecularly imprinted material provided 60% enhancement in enantioselectivity versus a nearly identical formulation using TRIM. Other molecularly imprinted materials using the crosslinkers PETRA and PETEA also showed little to no enantioselective performance, supporting the conclusion that the difunctional crosslinker EDMA is required for these systems versus any trifunctional crosslinkers.

## Figures and Tables

**Table 1:** Chromatographic retention (k’) and enantioselectivity (α) of various MIP-CSPs tested in slurry packed columns (S1–11).

#	T (mmol)	M(s) (mmol)	Me (mmol)	C (mmol)	Molar Ratio T:M:Me:C	A (mmol)	P (mL)	k’_S_/k’_R_	α
S1	S-ATNL (0.3)	NOBE (7.6)	-	-	1:25:-:-	-	DMF (4)	33.2/31.6	1.05 ^a^
S2	S-ATNL (0.3)	NOBE (7.6)	Cu(II) (0.3)	-	1:25:1:-	-	DMF (4)	33.2/31.6	1.05 ^a^
S3	S-ATNL (0.3)	NOBE (7.6)	Co(II) (0.3)	-	1:25:1:-	-	DMF (4)	36.8/36.3	1.01 ^a^
S4	S-ATNL (0.3)	NOBE/MAA (7.6/1)	-	-	1:25/3:-:-	-	ACN (2)	4.4/3.8	1.17 ^b^
S6	S-ATNL (0.3)	4-VPy (0.3)	Co(II) (0.3)	TRIM (7.3)	1:1:1:24	-	DMF/DMSO (2/2)	3.6/3.5	1.03 ^a^
S7	S-ATNL (0.3)	4-VPy (1.5)	Co(II) (0.3)	TRIM (6.1)	1:5:1:20	-	DMF/DMSO (2/2)	8.2/7.9	1.04 ^a^
S8	S-ATNL (0.3)	4-VPy (3)	Co(II) (0.3)	TRIM (4.6)	1:10:1:15	-	DMF/DMSO (2/2)	8.2/7.9	1.04 ^a^
S9	S-ATNL (0.3)	4-VPy/AM (0.3/1.2)	Co(II) (0.3)	TRIM (6.1)	1:1/4:1:20	-	DMF/DMSO (2/2)	0.5/0.5	1.00
S10	S-ATNL (0.3)	4-VPy/BAM (0.3/1.2)	Co(II) (0.3)	TRIM (6.1)	1:1/4:1:20	-	DMF/DMSO (2/2)	0.6/0.5	1.07 ^a^
S11	S-ATNL (0.15)	4-VPy (0.9)	Co(II) (0.15)	EDMA (3.6)	1:6:1:24	IL (1.235)	DMF/DMSO (0.12/0.6)	16.2/12.3	1.32 ^a^

Mobile phase composition: ^a^ ACN; ^b^ ACN:50 mM formate buffer, pH 3 = 86:14, *v*/*v*. In a typical thermal (60 °C) or photo-induced polymerization (24 h), the pre-polymerization mixture also includes 15 mg of AIBN as free radical initiator. T—template, M—monomer, Me—metal ion, C—cross-linker, A—additive, P—porogen, IL—[BMIM]BF_4_.

**Table 2:** Chromatographic retention (k’) and enantioselectivity (α) of various MIP-CSPs tested in monolithic columns (M1–10) with 4-VPy as functional monomer.

#	T (mmol)	M(s) (mmol)	Me (mmol)	C (mmol)	Molar Ratio T:M:Me:C	A (mmol)	P (mL)	k’_S_/k’_R_	α
M1	S-ATNL (0.2)	4-VPy (1)	Co(II) (0.2)	TRIM (6)	1:5:1:30	IL (0.9)	DMF/DMSO (0.9/0.9)	4.3/4.3	1.00
M2	S-ATNL (0.15)	4-VPy (0.9)	Co(II) (0.15)	EDMA (3.6)	1:6:1:24	IL (1.235)	DMF/DMSO (0.12/0.6)	3.7/2.3	1.60 ^a^
M3	S-ATNL (0.15)	4-VPy (0.9)	Ni(II) (0.15)	EDMA (3.6)	1:6:1:24	IL (1.235)	DMF/DMSO (0.12/0.6)	9.4/6.8	1.38 ^a^
M4	S-ATNL (0.15)	4-VPy (0.9)	Cu(II) (0.15)	EDMA (3.6)	1:6:1:24	IL (1.235)	DMF/DMSO (0.48/0.6)	6.8/5.3	1.30 ^a^
M5	S-ATNL (0.15)	4-VPy (0.9)	Co(II) (0.15)	EDMA (3.6)	1:6:1:24	- *	DMF/DMSO (0.326/1.630)	6.3/5.8	1.08 ^a^
M6	S-ATNL (0.15)	4-VPy (0.9)	-	EDMA (3.6)	1:6:-:24	IL (1.235)	DMF/DMSO (0.12/0.6)	3.4/2.6	1.30 ^a^
M7	S-ATNL (0.15)	4-VPy (0.9)	Co(II) (0.15)	EDMA (3.6)	1:6:1:24	IL/PEGMA (1.235/0.17)	DMF/DMSO (0.12/0.6)	4.0/4.0	1.00
M8	-	4-VPy (0.9)	Co(II) (0.15)	EDMA (3.6)	-:6:1:24	IL (1.235)	DMF/DMSO (0.12/0.6)	2.7/2.6	1.04 ^a^
M9	-	4-VPy (0.9)	-	EDMA (3.6)	-:6:-:24	IL (1.235)	DMF/DMSO (0.12/0.6)	10.4/10.4	1.00
M10	S-ATNL (0.05)	4-VPy (0.15)	Ni(II) (0.05)	TRIM (1.15)	1:3:1:23	-	MeOH (2)	0.3/0.3	1.00

Mobile phase composition: ^a^ ACN. * Replaced with the corresponding volume of DMF/DMSO 1:5, *v*/*v*. In a typical thermal (60 °C) or photo-induced polymerization (24 h), the pre-polymerization mixture also includes 15 mg of AIBN as free radical initiator. T—template, M—monomer, Me—metal ion, C—cross-linker, A—additive, P—porogen, IL—[BMIM]BF_4_.

**Table 3:** Chromatographic retention (k’) and enantioselectivity (α) of various MIP-CSPs tested in monolithic columns with 4-VPy and various co-monomers (M11–13), or different functional monomers, other than 4-VPy (M14–24).

#	T (mmol)	M(s) (mmol)	Me (mmol)	C (mmol)	Molar Ratio T:M:Me:C	A (mmol)	P (mL)	k’_S_/k’_R_	α
M11	S-ATNL (0.15)	4-VPy/AM (0.15/0.75)	Co(II) (0.15)	EDMA (3.6)	1:1/5:1:24	IL (1.235)	DMF/DMSO (0.12/0.6)	12.9/10.0	1.29 ^a^
M12	S-ATNL (0.15)	4-VPy/4-PBA (0.9/0.15)	Co(II) (0.15)	EDMA (3.6)	1:6/1:1:24	IL (1.235)	DMF/DMSO (0.12/0.6)	4.6/4.4	1.04 ^a^
M13	S-ATNL (0.15)	4-VPy/ CVPBA (0.9/0.15)	Co(II) (0.15)	EDMA (3.6)	1:6/1:1:24	IL (1.235)	DMF/DMSO (0.12/0.6)	0.4/0.4	1.00
M14	S-ATNL (0.15)	1-VIM (0.9)	Co(II) (0.15)	EDMA (3.6)	1:6:1:24	IL (1.235)	DMF/DMSO (0.12/0.6)	6.2/4.3	1.43 ^a^
M15	S-ATNL (0.15)	MAA/AM (0.15/0.75)	Cu(II) (0.15)	EDMA (3.6)	1:1/5:1:24	IL (1.235)	DMF/DMSO (0.12/0.6)	2.5/2.2	1.14 ^b^
M16	S-ATNL (0.15)	MAA/AM (0.15/0.75)	-	EDMA (3.6)	1:1/5:-:24	IL (1.235)	DMF/DMSO (0.12/0.6)	15.3/14.5	1.06 ^b^
M17	S-ATNL (0.13)	MAA (0.7)	-	EDMA (0.7)	1:5:-:5	-	ACN (2)	1.8/1.5	1.15 ^c^
M18	S-ATNL (0.13)	MAA (0.7)	-	EDMA (0.7)	1:5:-:5	IL (1.235)	ACN (0.72)	6.2/4.7	1.32 ^d^
M19	S-ATNL (0.13)	MAA (0.7)	-	PETRA(0.7)	1:5:-:5	IL (1.235)	ACN (0.72)	8.7/7.8	1.11 ^e^
M20	S-ATNL (0.2)	VFC (0.1)	-	TRIM (1.25)	2:1:-:12.5	-	ACN (1%H_2_O) (5)	0.3/0.3	1.00
M21	S-ATNL (0.2)	1-VIM (0.6)	Ni(II) (0.2)	TRIM (1.25)	1:3:1:6	-	MeOH (5)	0.9/0.9	1.00
M22	S-ATNL (0.04)	1-VIM (0.04)	Ni(II) (0.04)	PETEA (0.25)	1:1:1:6	-	MeOH (1)	0.5/0.5	1.00
M23	S-ATNL (0.02)	1-VIM/BAM (0.02/0.08)	Cu(II) (0.02)	-	1:1/4:1:-	-	MeOH	2.4/2.4	1.00
M24	S-ATNL (0.2)	1-VIM/MAA (0.6/0.2)	Cu(II) (0.2)	PETRA (3.6)	1:3/1:1:18	-	BuOH (5)	3.6/3.5	1.03 ^c^

Mobile phase composition: ^a^ ACN; ^b^ ACN:50 mM formate buffer, pH 3 = 95:5, *v*/*v*; ^c^ ACN:50 mM formate buffer, pH 3 = 90:10, *v*/*v*; ^d^ ACN:50 mM formate buffer, pH 3 = 80:20, *v*/*v*; ^e^ ACN:50 mM acetate buffer, pH 5 = 80:20, *v*/*v*. In a typical thermal (60 °C) or photo-induced polymerization (24 h), the pre-polymerization mixture also includes 15 mg of AIBN as free radical initiator. T—template, M—monomer, Me—metal ion, C—cross-linker, A—additive, P—porogen, IL—[BMIM]BF_4_.
